# GerenciaDOR™: development of digital technology by nurses for the assessment of patients with chronic pain

**DOI:** 10.1590/0034-7167-2024-0050

**Published:** 2024-12-16

**Authors:** Bianca Ferreira da Silva Brandão, Eliseth Ribeiro Leão

**Affiliations:** I Faculdade Israelita de Ciências da Saúde Albert Einstein. São Paulo, São Paulo, Brazil; II Instituto de Ensino e Pesquisa Albert Einstein, Hospital Israelita Albert Einstein. São Paulo, São Paulo, Brazil

**Keywords:** Chronic Pain, Digital Technology, Mobile Applications, Pain Measurement, Nurses., Dolor Crónico, Tecnología Digital, Aplicaciones Móviles, Dimensión del Dolor, Enfermeras.

## Abstract

**Objectives:**

to develop a digital technological solution (prototype) for assessing patients with chronic pain.

**Methods:**

this is a methodological and technological development study based on the Human-Centered Design framework and the principles of Patient-Centered Care. The prototype guides patients through a body diagram and directs them to an evaluation using specific instruments that address the multidimensional aspects of chronic pain.

**Results:**

the GerenciaDOR* project enables navigation through the Web App screens, providing access to pain assessment features up to the presentation of results.

**Final Considerations:**

the study describes a systematic approach to pain assessment and expands nurses’ knowledge in pain management. Additionally, it can promote the development of other digital technologies for chronic pain assessment and contribute to a multidisciplinary, patient centered treatment.

## RESUMEN

**Objetivos:**

desarrollar una solución tecnológica digital (prototipo) para la evaluación de pacientes con dolor crónico.

**Métodos:**

estudio metodológico y de desarrollo tecnológico basado en el marco de Diseño Centrado en el Ser Humano y los principios del Cuidado Centrado en el Paciente. El prototipo guía a los pacientes mediante un diagrama corporal y los dirige a una evaluación con instrumentos específicos que abordan los aspectos multidimensionales del dolor crónico.

**Resultados:**

el proyecto GerenciaDOR™ permite la navegación a través de las pantallas del Web App, proporcionando acceso a las funciones de evaluación del dolor hasta la presentación de resultados.

**Consideraciones Finales:**

el estudio describe un enfoque sistemático para la evaluación del dolor y amplía el conocimiento de los enfermeros en el manejo del dolor. Además, puede promover el desarrollo de otras tecnologías digitales para la evaluación del dolor crónico y contribuir a un tratamiento multidisciplinario centrado en el paciente.

## INTRODUCTION

Acute pain serves as a warning signal for the body, indicating something is wrong and requires investigation. It differs from chronic pain, which is recognized as a disease and may persist even after an injury has healed. Managing chronic pain requires a multidisciplinary approach due to its biological, psychological, social, emotional, cultural, and even spiritual dimensions. Although pain relief has been recognized as a human right by the Montreal Declaration since 2010^(1,2)^, chronic pain remains undertreated and neglected by many healthcare professionals.

In Brazil, an estimated 40% of the population suffers from chronic pain, and its management is often inadequate, partly because its multidimensional nature is not routinely considered in assessment and treatment. Pain evaluation should be regarded as a central clinical competency, often seen as challenging due to its subjective nature and the complex concepts involved in its understanding. Accurate and standardized assessment is crucial for advancing research, developing new therapies, and formulating effective and safe treatment plans. Moreover, reliable data can influence public health policies and resource allocation, making treatments more accessible and optimizing the limited time available during consultations^(3,4)^.

In the national context, only one app that utilizes specific assessment tools has been identified. It aims to provide a more accurate diagnosis within 20 minutes for a multidisciplinary team. However, no studies have demonstrated the outcomes of this product’s use so far. Another challenge is that access to the app requires payment, limiting its use even for testing purposes.

Given the complexity of pain management and the abundance of information involved, there is a need for a technology that can help organize assessment stages and standardize data based on scientific evidence. Such technology should consider each patient’s individual needs, allow continuous monitoring of their progress by the entire care team, and facilitate more accurate decision making^(3-5)^. These aspects of patient education, care, and monitoring in chronic pain management motivated this study, resulting from a master’s thesis. The study focused on developing a new technology for pain assessment, an essential first step in guiding the treatment of chronic pain patients and providing personalized care.

## OBJECTIVES

To develop a digital technological solution (prototype) for assessing patients with chronic pain.

## METHODS

This study, conducted from April to November 2023, involved developing a technological prototype of a Web App. It was a collaboration between the authors-experienced nurses in pain management, education, and research-and information systems professionals with over ten years of experience in the healthcare digital marketing sector.

From the search for the ideal developer to the completion of the product, the process took approximately ten months, involving virtual meetings between a nurse and the company’s executive director of innovations. They discussed the need for a technology capable of assessing chronic pain in patients, incorporating three main components:

1. An app for patients and healthcare professionals to monitor pain and medication use, respond to specific evaluation questionnaires, and visualize insights into their conditions;
2. Reports summarizing the app’s collected data to be used prior to nursing and medical consultations, enhancing communication among patients and the multidisciplinary team, and monitoring periodic responses to proposed treatments;
3. A monitoring portal for clinics to remotely evaluate patient progress, identify clinically relevant trends and patterns using advanced analytics, and facilitate data aggregation for research and publications.

After confirming the feasibility of incorporating all the requirements, the study was anchored in the Human-Centered Design framework, which provides tools and concepts for developing innovative, effective, sustainable, and creative solutions. This methodology places the human being, along with their problems and needs, at the center of attention; includes practices where the patient and their family actively participate in decision-making related to all aspects of their health care; ensures personalized and holistic care that aligns with the individual’s preferences and demands; and encourages the patient to take an active role in their own care^(6,7)^.

The GerenciaDOR project aims to compile all relevant patient history data on pain complaints. It employs visual strategies and eight specific assessment tools (Chart 1) to guide the development of treatment plans and ongoing care.

**Chart 1:** Assessment tools selected for GerenciaDOR

Assessment Tools	Target Population
Brief Pain Inventory (BPI)^(8)^	All patients attending initial consultations and following periodic treatment
Short Form Health Survey (SF-36)^(9)^	All patients attending initial consultations and following periodic treatment
Neck Disability Index (NDI^)(10^)	Patients with neck pain and periodic follow-up
Disabilities of Arms, Shoulder and Hands (Quick DASH)^(11)^	Patients with shoulder and upper limb pain and periodic follow-up
Low Back Pain Disability Questionnaire OSWESTRY (ODI)^(12)^	Patients with low back pain and periodic follow-up
Western Ontario and MacMaster (WOMAC)^(13)^	Patients with hip and knee pain and periodic follow-up
Neuropathic Pain Assessment (DN4)^(14)^	Referred for neuropathic pain diagnosis
Headache Impact Test (HIT-6)^(15)^	Assessment of patients with headaches and periodic follow-up

*ODI - OSWESTRY disability index; DN4 - Douleur neuropathique.*

**Figure 1:**
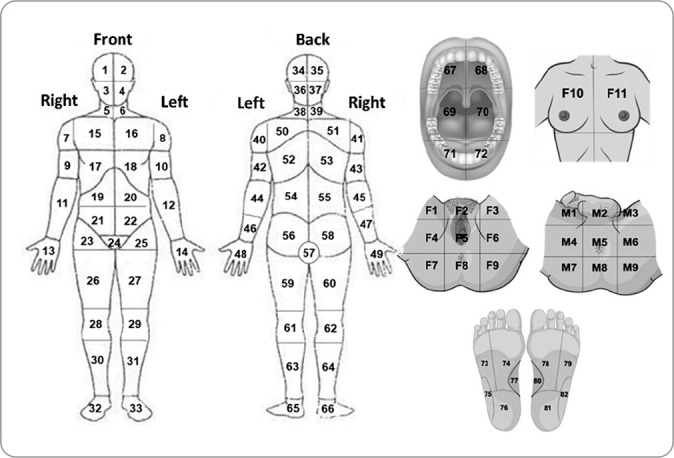
Body diagram adapted from Cleeland & Ryan (1994) and GerenciaDOR’s unique features

One of GerenciaDOR’s distinguishing features is its comprehensive approach to addressing the specific details of the pain areas reported by patients, including regions that are not covered by any existing assessment instruments in the literature (Figure 1).

To achieve this, the application provides a customized body diagram. Patients use this diagram to indicate each selected area’s frequency, intensity, and pain characteristics and to respond to the relevant assessment questionnaires. A periodic follow-up will be suggested through links, allowing comparison of results and monitoring of the treatment progress post-consultation. After completing all stages of the pain assessment, the patient will undergo the nursing consultation (NC). The goal of GerenciaDOR is not only to reduce the time needed for assessment but also to provide the nurse with a preview of the patient’s complaint. This will enable the nurse to better direct their questions while collecting the patient’s history in the NC, guided by a structured protocol consisting of key inquiries necessary to compose the patient’s anamnesis.

## RESULTS

**Figure 2:**
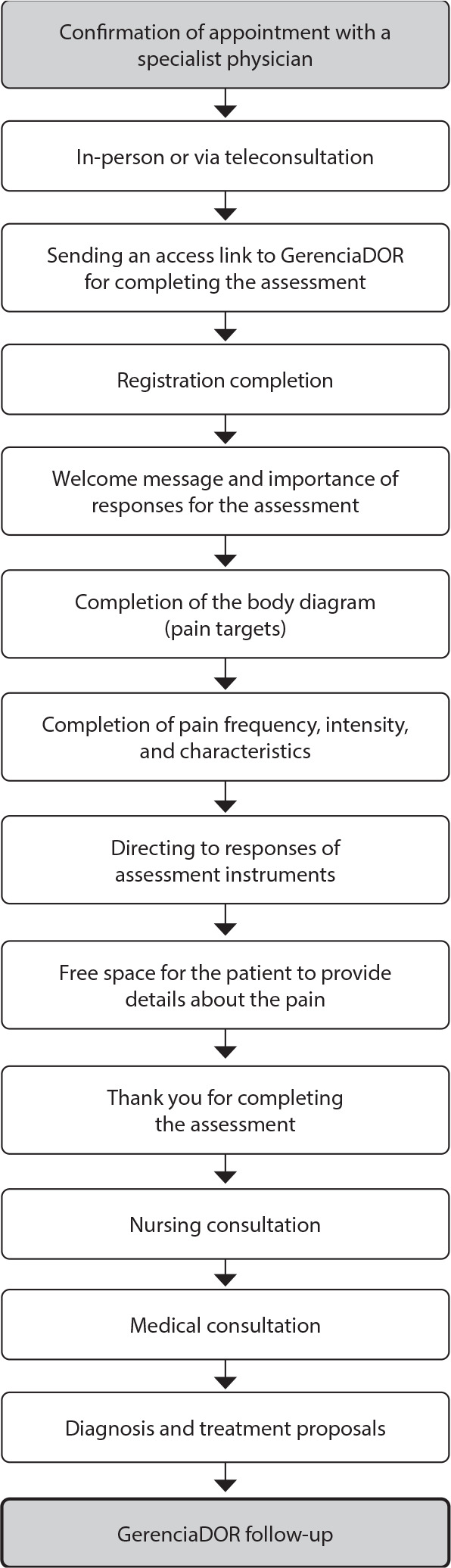
Representation of GerenciaDOR front-ends

Once the appointment is scheduled, the patient will follow the evaluation journey as illustrated in the flowchart in Figure 2, representing the logic developed in the prototype.

**Figure 3:**
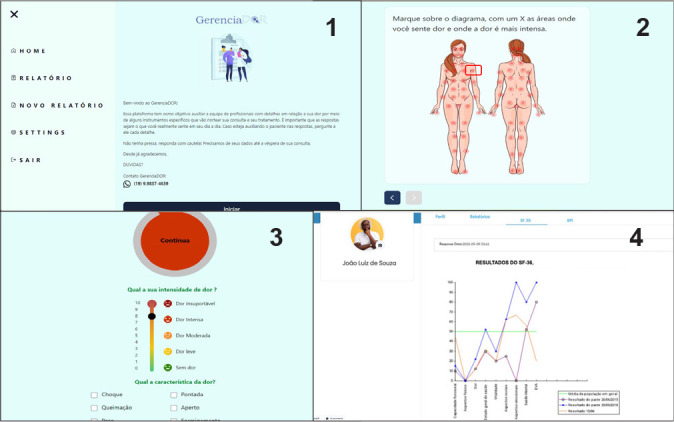
Representation of GerenciaDOR front-ends

The platform is further represented by the sequence of front-end screens (Figure 3). The *front end*, a term used in the field of information technology, refers to the part of the application visible to and interacted with by the user, such as colors, fonts, menus, and images, among other functionalities. The reproduced screens are from the Web App accessed via a computer but remain consistent across different devices (e.g., mobile phones).

Translation: Chart 1 on the left (from top to bottom): Home, Report, New Report, Settings, Exit. On the right (from top to bottom): Welcome to GerenciaDOR! This platform aims to help the team of professionals with details regarding your pain through some specific instruments that will guide your consultation and treatment. It’s important that the answers are really what you feel in your daily life. If you’re helping the patient with answers, ask him for every detail. Take your time, respond carefully. We need your details by the day before your appointment. Thank you in advance. Doubts? Contact GerenciaDOR (19) 99827-4639; Chart 2: Mark on the diagram with an X the areas where you feel pain and where the pain is most severe; Chart 3: Choose the circle that best represents the frequency of your pain. How intense is your pain? Unbearable pain, Intense pain, Moderate pain, Mild pain, No pain. What is the characteristic of the pain? Shock, Sting, Burning, Grip, Tingling, Others; Chart 4: Graphical representation of instruments throughout the treatment.

Clockwise, the front-ends in Figure 3 represent: 1) welcome and menu screens, 2) targets on the female body diagram for directing responses to assessment instruments, 3) response screen for pain frequency, intensity, and characteristics, and 4) screen for displaying results and follow-up with a trend graph of comparative results (fictitious patient) according to the proposed treatment plan.

## DISCUSSION

GerenciaDOR aims to provide a technological solution for regularly and effectively assessing and monitoring patients with chronic pain. Inconsistent and inadequate assessments can impact and limit therapeutic proposals; this project, therefore, becomes an ally in organizing priorities amid the many demands inherent to the nurse’s role^(16)^.

Thoroughly obtaining a pain history, examining the patient, and using various tests and questionnaires to assess pain is essential but only the first step. One of the most common parameters for evaluation is pain intensity, which denotes the strength of the subjective experience (“How much does it hurt?”). Typically, it is assessed using self-report scales such as the Visual Analog Scale (VAS) and the Numeric Rating Scale (NRS), tools commonly applied for evaluating this pain parameter in clinical studies and medical settings. These subjective methods have been considered the gold standard for assessing pain. However, their accuracy and utility are limited in certain circumstances, as patients must be conscious and oriented in time and space, free from the effects of sedatives and/or anesthetics, and any cognitive impairment; they must also possess basic literacy skills.

Carefully evaluating the outcomes reported by patients and obtained through the Web App encompasses pain domains, quality of life, functional impairment in performing activities of daily living, socio-emotional aspects, and other dimensions. This comprehensive approach is necessary to capture the multidimensionality of the overall chronic pain experience, helping guide treatment decisions and increasing patient engagement in their own care. For this purpose, GerenciaDOR will utilize follow-up link strategies at predetermined times according to the established treatment plan, enabling comparison with pre-consultation results.

This array of challenges and unique contexts requires healthcare professionals involved in patient pain management to adopt scientifically grounded practices. They must also acquire the ability to convey this knowledge clearly and accessibly to the patient, with effective records that the entire multidisciplinary team can access. Moreover, choosing appropriate instruments that represent the population served in each type of service is crucial.

The potential for developing applications tends to increase as the population ages and the need for remote communication grows. More than that, technological alternatives provide a personalized, efficient, cost-effective environment and an intelligent experience for pain management. In this context, the number of mobile applications in the United States and worldwide is increasing to refine pain management. However, many of them fall short of expectations, as neither physicians nor other healthcare professionals were involved in developing the content, resulting in deficiencies due to a lack of scientific basis.

Studies confirm that the trend of remote and digital follow-up offers an alternative to in-person follow-up, which is often timid and restricted, and may lead to issues due to the loss of pain variable parameters over time. There is a consensus that chronic pain requires a multidisciplinary approach, but not just that: well-structured and organized multidisciplinary programs demonstrate greater efficacy. It all begins with robust communication among team members, active patient participation, self-management strategies, and a care flow-as stated in a Canadian study that also relies on PCC and evidence-based processes supported by a government program. Initiatives like this educate patients and healthcare professionals, demonstrate continuous quality improvement in care, provide data information, promote exchange and research, and offer access to continuous care.

PCC care and communication can be facilitated in various ways, including building strong relationships between nurses and patients. This establishes active listening, which is essential to addressing barriers to care. By listening to patients and their concerns, nurses can identify their needs and preferences and address their fears and frustrations^(7)^.

Effective pain management is an important and sensitive indicator of the quality of nursing and healthcare services. While achieving satisfactory pain relief requires interdisciplinary collaboration and is an ethical and moral responsibility of all professionals involved in care, nurses play an essential role in alleviating patient pain as experts in pain assessment, management, and education at all levels of care and healthcare settings. This study, conducted by nurses, contributes to legitimizing the need for other colleagues to empower themselves with knowledge in the field of pain management, a specialty still underexplored in the market. This demand is confirmed by Al-Sayagh and team in a cross-sectional study conducted in Saudi Arabia, after evaluating over 600 professionals in various hospital and outpatient departments. They acknowledged that, although satisfactory pain reduction requires interdisciplinary team collaboration, nurses, as specialists in pain assessment, treatment, and education across all healthcare settings, play a crucial role in relieving patient pain through up-to-date knowledge and appropriate skills. Digital pain management apps and other health-related clinical applications deserve significant attention in the coming years, given the shift toward mobile health tools and telemedicine, which expands nurses’ scope into this new area^(5,6)^.

### Study limitations

One of the primary limitations of this project was the lack of access to a database for GerenciaDOR, even though this is just a prototype. This investment is crucial, as without this functionality, users cannot request help for password recovery since their registration information would not be stored. Regarding completing the body diagram, patients can select only one affected area at a time, which prevents the visualization of multiple affected sites simultaneously. This issue can be addressed during the nurse’s initial interaction with the patient, as the information will be confirmed and compiled. However, it is essential to fully understand the nature of the complaints before this interaction to better guide the questions and conduct the Nursing Consultation.

It is also worth noting the inability to validate the content with the end user, whether the patient or another healthcare professional. Nonetheless, this will be the focus of future development stages until its implementation in clinical practice, at which point all material will be linked to a database. This procedure will enable more consistent validation with the actual functionalities the Web App will offer. Additionally, future stages aim to incorporate new features into the digital platform to enable comprehensive, interactive, and intuitive monitoring and to better understand how patients will integrate GerenciaDOR into their follow-up routines.

### Contributions to Nursing

Despite the limitations above, completing this stage of the GerenciaDOR project provides valuable insights, directs opportunities for developing mobile solutions that support chronic pain management in outpatient settings, and allows for adaptation to hospital settings by including appropriate instruments. In an increasingly digital world, trends indicate that nurses must develop technological strategies for pain management to engage users with the proposed interventions, regardless of the chosen resource modality, as long as it is accessible to patients. These strategies should be explored as alternatives to enable nurses to assess pain and establish effective communication with their patients, even in remote areas. In this regard, there is substantial evidence of the effectiveness of telehealth interventions for addressing pain-related issues.

The GerenciaDOR project combined the need to gather detailed data from assessing patients with chronic pain with the technological ease of compiling information and enabling better follow up during treatment. This is achieved through real-time access to the patient’s history by the healthcare team, patient, family member, or caregiver at any time, from any location and device. Additionally, the project optimizes the patient’s time after arriving at the clinic, as part of the assessment will have been completed in advance. The healthcare professional can use the time saved to strengthen the patient-provider relationship and address diagnostic and treatment related issues.

In Brazil, the future of pain management depends on conducting new research to understand the factors that influence the initial acceptance and ongoing engagement with digital applications or technologies for assessing patients with pain. This is crucial because all the studies cited reflect the reality of developed countries, which differs from the national healthcare context.

## FINAL CONSIDERATIONS

This study was based on scientific literature and the extensive experience of the authors to develop a prototype for the GerenciaDOR Project’s pain assessment, intended for a specialized chronic pain service. It highlights the importance of nurses’ roles in this field and may inspire the development of new digital technologies.

## References

[1] Cohen SP, Vase L, Hooten WM. (2021). Chronic pain: an update on burden, best practices, and new advances. Lancet.

[2] International Association for the Study of Pain (IASP) (2021). Access to pain management: declaration of Montreal.

[3] Joypaul S, Kelly F, McMillan SS, King MA. (2019). Multi-disciplinary interventions for chronic pain involving education: a systematic review. PLoS One.

[4] Gebke KB, McCarberg B, Shaw E, Turk DC, Wright WL, Semel D. (2023). A practical guide to recognize, assess, treat and evaluate (RATE) primary care patients with chronic pain. Postgrad Med.

[5] Zhao P, Yoo I, Lancey R, Varghese E. (2019). Mobile applications for pain management: an app analysis for clinical usage. BMC Med Inform Decis Mak.

[6] Rossi E, Di Nicolantonio M. (2020). Integrating human-centred design approach into Sustainable-Oriented 3D Printing Systems. Hum Intell Syst Integr.

[7] The Health Foudation (UK) (2016). Person-centred care made simple: what everyone should know about person-centred care.

[8] Cleeland CS, Ryan KM. (1994). Pain assessment: global use of the Brief Pain Inventory. Ann Acad Med Singap.

[9] Ware JE, Sherbourne CD. (1992). The MOS 36-item short-form health survey (SF-36): conceptual framework and item selection. Med Care.

[10] Vernon H, Mior S. (1991). The Neck Disability Index: a study of reliability and validity [1] [:followi]. J Manipulative Physiol Ther.

[11] Hudak PL, Amadio PC, Bombardier C. (1996). Development of an upper extremity outcome measure: the DASH (disabilities of the arm, shoulder and hand) [Corrected]. The Upper Extremity Collaborative Group (UECG). Am J Ind Med.

[12] Fairbank JC, Couper J, Davies JB, O’Brien JP. (1980). The Oswestry low back pain disability questionnaire. Physiotherap.

[13] Bellamy N, Buchanan WW, Goldsmith CH, Campbell J, Stitt LW. (1988). Validation study of WOMAC: a health status instrument for measuring clinically important patient relevant outcomes to antirheumatic drug therapy in patients with osteoarthritis of the hip or knee. J Rheumatol.

[14] Bouhassira D, Attal N, Alchaar H, Boureau F, Brochet B, Bruxelle J (2005). Comparison of pain syndromes associated with nervous or somatic lesions and development of a new neuropathic pain diagnostic questionnaire (DN4). Pain.

[15] Kosinski M, Bayliss MS, Bjorner JB, Ware JE, Garber WH, Batenhorst A (2003). A six-item short-form survey for measuring headache impact: the HIT-6. Qual Life Res.

[16] Suso-Ribera C, Castilla D, Zaragozá I, Mesas Á, Server A, Medel J (2020). Telemonitoring in chronic pain management using smartphone apps: a randomized controlled trial comparing usual assessment against app-based monitoring with and without clinical alarms. Int J Environ Res Public Health.

