# Supplementary material for: GerenciaDOR™: development of digital technology by nurses for the assessment of patients with chronic pain
Source: Rev Bras Enferm. 2024 Dec 16;77(6):e20240050. doi: 10.1590/0034-7167-2024-0050 (PMC11654545; doi:10.1590/0034-7167-2024-0050)
Supplement: Translation

# Translation: GerenciaDOR™: desenvolvimento de tecnologia digital por enfermeiras para avaliação de pacientes com dores crônicas

**DOI:** 10.1590/0034-7167-2024-0050pt

**Authors:** Bianca Ferreira da Silva Brandão, Eliseth Ribeiro Leão

**Affiliations:** I Faculdade Israelita de Ciências da Saúde Albert Einstein. São Paulo, São Paulo, Brasil; II Instituto de Ensino e Pesquisa Albert Einstein, Hospital Israelita Albert Einstein. São Paulo, São Paulo, Brasil

**Keywords:** Dor Crônica, Tecnologia Digital, Aplicativos Móveis, Mensuração da Dor, Enfermeiras.

## Abstract

**Objetivos:**

desenvolver uma solução tecnológica digital (protótipo) para avaliação de pacientes com dores crônicas.

**Métodos:**

trata-se de um estudo metodológico e de produção tecnológica para avaliação da dor crônica seguindo o referencial denominado *Human-Centered* e os princípios do Cuidado Centrado no Paciente. O protótipo permite que o paciente seja guiado por um diagrama corporal e direcionado para avaliação composta por instrumentos específicos abordando aspectos multidimensionais da dor crônica.

**Resultados:**

o GerenciaDOR possibilita a navegação pelas telas do Web App, com acesso às funcionalidades disponíveis para avaliação da dor até a fase de apresentação de resultados.

**Considerações Finais:**

o estudo apresenta uma avaliação da dor sistematizada e contribui com a ampliação do conhecimento dos enfermeiros na área de manejo da dor. Além disso, pode impulsionar o desenvolvimento de outras tecnologias digitais para avaliação da dor crônica e contribuir com um tratamento multidisciplinar qualificado e direcionado às necessidades do paciente.

## INTRODUÇÃO

A dor aguda tem uma função de alerta para nosso organismo, sinalizando que algo está errado e merece investigação. Difere da dor crônica, que é reconhecida como uma doença e pode persistir mesmo após a cura de uma lesão. Ela exige abordagem de uma equipe multidisciplinar, pois envolve aspectos biológicos, psicológicos, sociais, emocionais, culturais e até mesmo espirituais. Representa um dos principais motivos de busca por atendimento médico; apesar disso, é subtratada e negligenciada pelas diversas categorias de profissionais da área, ainda que o alívio de dor seja assegurado como um direito humano pela Declaração de Montreal desde 2010^(1,2)^.

No Brasil, estima-se que cerca de 40% da população sofra com dores crônicas e que seu manejo seja inadequado, em parte devido à multidimensionalidade, a qual não é incorporada rotineiramente em sua avaliação e tratamento. A avaliação da dor deve ser vista como uma capacidade clínica central, muitas vezes considerada difícil, talvez em razão da sua natureza subjetiva e complexidade de conceitos envolvidos para sua compreensão. Em termos gerais, uma avaliação precisa e padronizada é crucial para o desenvolvimento de novas pesquisas e vital para o avanço do conhecimento, surgimento de novas terapias e formulação de planos de tratamento eficazes e seguros. Além disso, dados criteriosos e confiáveis podem influenciar a criação de políticas de saúde pública e alocação de recursos, inclusive para tratamentos mais acessíveis, bem como otimizar o tempo muitas vezes limitado para as consultas^(3,4)^.

No cenário nacional, identificamos apenas um aplicativo que utiliza instrumentos específicos de avaliação. Um dos objetivos é um diagnóstico mais assertivo, disponibilizado para equipe multiprofissional em até 20 minutos, porém não há estudos demonstrando os resultados com o uso do produto até o momento. Outra dificuldade é que o acesso ao aplicativo está vinculado a um pagamento, o que restringe o uso mesmo que seja somente para testes.

Diante da complexidade no gerenciamento da dor e da gama de informações envolvidas, é conveniente uma tecnologia que possa colaborar na organização das etapas de avaliação e na padronização dos dados, com embasamento científico. Essa tecnologia deve considerar as necessidades individuais de cada paciente, permitir o monitoramento contínuo de sua evolução por toda a equipe de cuidados e favorecer a tomada de decisões mais assertiva^(3-5)^. Todos esses aspectos que abarcam a educação, cuidado e monitoramento do paciente com dor crônica motivaram a realização deste trabalho, fruto de uma dissertação de mestrado. O estudo foi dedicado ao desenvolvimento de nova tecnologia para avaliação da dor, etapa inicial e essencial para direcionar o tratamento de pacientes com dores crônicas e oferecer um atendimento personalizado.

## OBJETIVOS

Desenvolver uma solução tecnológica digital (protótipo) para avaliação de pacientes com dores crônicas.

## MÉTODOS

Trata-se de uma pesquisa de produção tecnológica (protótipo), em formato de *Web App*, desenvolvida entre abril e novembro de 2023. Foi realizada em cooperação com enfermeiras, autoras deste estudo, com ampla experiência em gestão, educação e pesquisa na área da dor, junto com profissionais de sistemas de informação com experiência de mais de dez anos no mercado especializado em marketing digital na área da saúde.

Desde a busca pelo desenvolvedor ideal até a conclusão do produto, foram aproximadamente dez meses de trabalho. As inúmeras reuniões sempre foram realizadas virtualmente pela enfermeira junto com o diretor executivo de inovações da empresa, expondo a necessidade de criar uma tecnologia para avaliar a dor de pacientes com dores crônicas. Ela deveria englobar três componentes:

1. Um aplicativo para pacientes e profissionais de saúde para monitorar a dor e medicamentos em uso; responder a questionários específicos de avaliação; e visualizar insights sobre suas condições;
2. Relatórios que resumissem as informações coletadas no aplicativo para serem usados antes das consultas de enfermagem (CE) e consultas médicas, a fim de facilitar a comunicação entre pacientes e profissionais da equipe multiprofissional e de monitorar respostas periódicas ao(s) tratamento(s) proposto(s);
3. Um portal de monitoramento usado pelas clínicas para avaliar remotamente o progresso de seus pacientes e identificar tendências clinicamente relevantes e padrões usando análises avançadas, facilitando o agrupamento de dados para estudos e publicações na área.

Após afirmação da possibilidade de agrupar todos os requisitos, o estudo foi ancorado pelo referencial *Human-Centered Design,* que disponibiliza ferramentas e conceitos para projetos de soluções inovadoras, eficazes, sustentáveis e criativas. A metodologia consiste em colocar o ser humano, seus problemas e suas necessidades como o centro das atenções, tendo como aliado o Cuidado Centrado no Paciente (CCP); inclui práticas em que o paciente e seus familiares participam ativamente das decisões que envolvem toda a assistência relacionada à sua saúde; assegura um atendimento personalizado e holístico de acordo com as vontades e demandas individuais; e encoraja o paciente a assumir um papel ativo em seus cuidados^(6,7)^.

O projeto GerenciaDOR intenciona compilar todos os dados pertinentes do histórico do paciente quanto às queixas de dor. Para isso, usa estratégias visuais e oito instrumentos de avaliação específicos (Quadro 1), que nortearão as condutas para seu plano de tratamento e continuidade de cuidados:

**Quadro 1:** Instrumentos de avaliação selecionados para compor o GerenciaDOR

Instrumentos de avaliação	Público-alvo
Inventário Breve de Dor (BPI)^(8)^	Todos os pacientes que passam em primeira consulta e seguem acompanhamento (*follow-up*) periódico do tratamento proposto
Questionário de qualidade de vida SF-36^(9)^	Todos os pacientes que passam em primeira consulta e seguem acompanhamento periódico do tratamento proposto
*Neck Disability Index* (NDI^)(10^)	Pacientes com cervicalgia e acompanhamento periódico
*Disabilities of Arms, Shoulder and Hands (Quick DASH*)^(11)^	Pacientes com dores nos ombros e MMSS e acompanhamento periódico
Índice de incapacidade para avaliação de lombalgia OSWESTRY (ODI)^(12)^	Pacientes com lombalgia e acompanhamento periódico
*Western Ontario and MacMaster* (WOMAC)^(13)^	Pacientes com dores no quadril e joelho e acompanhamento periódico
Avaliação de dor neuropática (DN4)^(14)^	Encaminhado para diagnóstico de dor neuropática
Teste de impacto da dor de cabeça (HIT-6)^(15)^	Avaliação de pacientes com cefaleia e acompanhamento periódico

*BPI - Brief inventory of pain; SF-36 - Short-form health survey; ODI - OSWESTRY disability index; DN4 - Douleur neuropathique; HIT6 - Headache impact test.*

Um dos diferenciais do GerenciaDOR é abordar de forma ampla as minúcias das áreas de dor sobre as quais os pacientes possam apresentar queixas. Também expõe regiões não contempladas em nenhum instrumento de avaliação disponível na literatura (Figura 1).

**Figura 1:** Diagrama corporal adaptado de Cleeland & Ryan (1994) e diferenciais GerenciaDOR

Para isso, o aplicativo tem um diagrama corporal personalizado. Por meio das áreas sinalizadas nesse diagrama, o paciente responde sobre a frequência, intensidade e características de dor de cada alvo selecionado, bem como os questionários de avaliação pertinentes. Um acompanhamento periódico será proposto pelo envio de links, o que permitirá comparar resultados e verificar a evolução proporcionada pelo tratamento proposto depois da consulta. Após a obtenção das respostas de todas as etapas de avaliação da dor, o paciente fará a CE. O objetivo do GerenciaDOR não é só reduzir o tempo, mas também oferecer ao enfermeiro uma prévia da queixa do paciente. Com isso, ele direcionará melhor suas perguntas durante a coleta de dados do histórico do paciente na CE, guiada por um roteiro estruturado constituído por questionamentos fundamentais para compor sua anamnese.

## RESULTADOS

Uma vez agendada a consulta, o paciente passará pela jornada de avaliação conforme demonstrado no fluxograma da Figura 2, que representa a lógica desenvolvida no protótipo.

**Figura 2:** Fluxograma para acesso ao GerenciaDOR

A plataforma segue representada pela sequência de *front-ends* de algumas telas (Figura 3). O *front-end*, termo utilizado na área de tecnologia da informação, corresponde à parte em que os usuários conseguem ver e interagir, como cores, fontes, menus e imagens, entre outras funcionalidades. As telas reproduzidas são do acesso ao Web App pelo computador, mas elas são as mesmas quando usados outros tipos de dispositivo (p.ex., celular).

**Figura 3:** Representação de *front-ends* GerenciaDOR

Em sentido horário, os *front-ends* da Figura 3 representam respectivamente: 1) as telas de boas-vindas e menu, 2) alvos do diagrama corporal feminino para direcionamento das respostas de instrumentos de avaliação, 3) tela de respostas da frequência, intensidade e características da dor e 4) tela de projeção de resultados e acompanhamento (*follow-up*) com gráfico de tendência de resultados comparativos (paciente fictício) conforme a proposta de tratamento.

## DISCUSSÃO

O GerenciaDOR visa encontrar uma solução tecnológica para avaliar e monitorar os pacientes com dores crônicas de forma regular e eficaz, já que, se aleatórios e inadequados, esses procedimentos tendem a impactar e limitar as propostas terapêuticas; portanto, esse projeto mostra-se um aliado na organização das prioridades perante tantas demandas inerentes à função do enfermeiro^(16)^.

Ser minucioso na obtenção de uma história de dor, examinando o paciente e usando diferentes testes e questionários para avaliar a dor é essencial, mas é apenas o primeiro passo. Um dos parâmetros mais utilizados para avaliação é a intensidade, que denota a força da experiência subjetiva (“Quanto dói?”). Geralmente, ela é avaliada com auxílio de escalas de autorrelato, como a Escala Visual Analógica (EVA) e a Escala Verbal Numérica (EVN), ferramentas comumente aplicadas para avaliar esse parâmetro da dor em estudos clínicos e em ambientes médicos. Tais métodos subjetivos foram considerados padrão-ouro para avaliar a dor. No entanto, a precisão e a utilidade do autorrelato são limitadas em certas circunstâncias, visto que os pacientes devem estar conscientes e orientados em tempo e espaço, livres de efeitos de sedativos e/ou anestésicos e de qualquer tipo de comprometimento cognitivo; além do mais, devem ter alfabetização mínima consolidada.

A avaliação cuidadosa dos resultados relatados pelos pacientes e obtidos pelo *Web App* abrange domínios de dor, qualidade de vida, comprometimento funcional de executar atividades de vida diária, aspectos socioemocionais e outras dimensões. Tudo isso é necessário para capturar a multidimensionalidade da experiência global do paciente com dor crônica e útil para orientar as decisões de tratamento, além de aumentar o envolvimento do paciente em seu próprio cuidado. Para isso, o GerenciaDOR utilizará estratégias de envios de links para *follow-up* em tempos pré determinados de acordo com a proposta de tratamento estabelecida para comparar com os resultados antes da consulta.

Essa conjuntura de dificuldades e contextos peculiares exige que os profissionais de saúde envolvidos nos cuidados ao paciente com dor tenham condutas embasadas cientificamente. Além disso, é fundamental que adquiram a capacidade de transferir esse conhecimento de forma clara e acessível para o paciente, com registros eficazes que possam ser acessados por toda equipe multiprofissional. Também é preciso que escolham os instrumentos adequados, os quais representem a população atendida em cada tipo de serviço.

O potencial para o desenvolvimento de aplicativos tende a aumentar à medida que a população envelhece e a necessidade de comunicação remota avança. Mais do que isso, alternativas tecnológicas possibilitam oferecer um ambiente personalizado, eficiente, econômico e uma experiência inteligente para o gerenciamento da dor. Nesse sentido, cresce o número de aplicativos móveis nos Estados Unidos e no mundo a fim de refinar o modo de gerenciar a dor. Todavia, muitos deles estão aquém das expectativas, já que nem médicos nem outros profissionais de saúde participaram do desenvolvimento do conteúdo, o que gerou deficiências pela falta de embasamento científico.

Estudos confirmam que a tendência de um acompanhamento remoto e digital são alternativas ao seguimento presencial que ocorre de forma tímida e restrita, além de se tornar um problema pela perda dos parâmetros das variáveis da dor ao longo dos dias. Há um consenso de que a dor crônica requer abordagem multidisciplinar, mas não apenas isso: programas multidisciplinares bem estruturados e organizados demonstram maior eficácia. Tudo começa com uma comunicação robusta entre os membros da equipe, participação ativa do paciente, estratégias de autogestão e um fluxo de cuidados - como afirmado em estudo canadense, que também recorre ao CCP e a todos os processos baseados em evidências apoiados por um programa governamental. Iniciativas como esta proporcionam educação para pacientes e profissionais de saúde; demonstram melhoria contínua da qualidade de atendimento; fornecem informações de dados; promovem intercâmbio e pesquisa; e oferecem acesso ao cuidado contínuo.

O cuidado e a comunicação no CCP podem ser facilitados de diversas maneiras, incluindo a construção de relacionamentos sólidos entre enfermeiros e pacientes, estabelecendo a escuta ativa, que é essencial para abordar barreiras ao cuidado. Ao ouvir os pacientes e suas preocupações, os enfermeiros podem identificar as suas necessidades e preferências e lidar com seus medos e frustrações^(7)^.

O manejo eficaz da dor é um indicador importante e sensível da qualidade dos serviços de enfermagem e de saúde. Embora alcançar o alívio satisfatório da dor exija colaboração interdisciplinar e seja uma responsabilidade ética e moral de todos os profissionais envolvidos no cuidado, os enfermeiros exercem uma função essencial no alívio da dor do paciente enquanto especialistas na avaliação, gestão e educação da dor em todos os níveis de atendimento e ambientes de saúde. O presente estudo, conduzido por enfermeiras, contribui com a legitimação da necessidade de outros colegas de profissão se empoderarem sobre o conhecimento na área de atuação em dor e magnitude de mercado de uma especialidade ainda pouco explorada. Essa demanda é confirmada por AL-Sayagh e equipe, em estudo transversal conduzido na Arábia Saudita após avaliação de mais de 600 profissionais em diversos departamentos da área hospitalar e ambulatorial. Eles reconheceram que, embora a redução satisfatória da dor exija a colaboração de equipe interdisciplinar, os enfermeiros, como especialistas na avaliação, tratamento e educação da dor em todos os ambientes de saúde, desempenham um papel crucial no alívio da dor do paciente por meio de um conhecimento atualizado e habilidades adequadas. Aplicativos digitais de gerenciamento da dor e outras aplicações clínicas relacionadas à saúde merecem atenção significativa nos próximos anos, dado o impulso em direção a ferramentas móveis de saúde e telemedicina, que ampliam o campo de atuação dos enfermeiros para essa nova área^(5,6)^.

### Limitações do estudo

Uma das principais restrições para esse trabalho foi a falta de acesso a um banco de dados para o GerenciaDOR, ainda que esse projeto se trate apenas de um protótipo. Esse investimento é fundamental, pois, sem essa funcionalidade, o usuário não pode solicitar auxílio por esquecimento da senha, já que suas informações de cadastro não ficaram armazenadas. Quanto ao preenchimento do diagrama corporal, o paciente pode selecionar somente uma área acometida de cada vez, o que impede a percepção dos múltiplos locais afetados em única visualização. Esse problema poderá ser resolvido no primeiro contato do enfermeiro com o paciente, uma vez que as informações serão confirmadas e compiladas. Entretanto, é essencial entender integralmente a lógica das queixas antes dessa abordagem, para um melhor direcionamento das perguntas e da condução da Consulta de Enfermagem.

Vale também ressaltar a impossibilidade de validação do conteúdo pelo usuário final, seja paciente, seja outro profissional especialista da equipe de saúde. No entanto, isso será alvo das próximas etapas de desenvolvimento até sua implementação na prática assistencial, quando todo material será vinculado a um banco de dados. Esse procedimento permitirá uma legitimação mais coerente com as funcionalidades que realmente o Web App oferecerá. Também, pretende-se agregar novas funcionalidades à plataforma digital em etapas futuras, para possibilitar um monitoramento integral, interativo e intuitivo e entender melhor como os pacientes incorporarão o uso do GerenciaDOR em sua rotina de acompanhamento.

### Contribuições para área da enfermagem

Apesar das limitações mencionadas, a conclusão dessa etapa do projeto GerenciaDOR capacita o fornecimento de insights, direciona oportunidades para o desenvolvimento de soluções móveis que apoiem a gestão da dor crônica em pacientes ambulatoriais e permite a adaptação para o atendimento hospitalar com inclusão de instrumentos adequados. Em um mundo cada vez mais digital, as tendências apontam que os enfermeiros deverão desenvolver estratégias tecnológicas para o gerenciamento da dor voltadas ao envolvimento dos usuários com as intervenções propostas, seja qual for a modalidade do recurso escolhida, desde que seja acessível aos pacientes. Essas estratégias devem ser exploradas como alternativas para capacitar o enfermeiro a avaliar a dor e construir um diálogo vantajoso com seus pacientes até mesmo se estiverem em áreas remotas. Nesse sentido, enfatiza-se que são substanciais as evidências de eficácia das intervenções de telessaúde para tratar problemas relacionados à dor.

O projeto GerenciaDOR uniu a necessidade de contemplar os dados de uma avaliação pormenorizada do paciente com dor crônica à facilidade tecnológica de compilar as informações e permitir um melhor acompanhamento durante o tratamento. Isso ocorre por meio do acesso ao histórico em tempo real pela equipe, paciente, familiar ou cuidador a qualquer momento e de qualquer lugar e dispositivo. Além disso, o projeto permite a otimização do tempo do paciente após a chegada no consultório, já que terá preenchido parte de sua avaliação antecipadamente. O tempo economizado poderá ser usado pelo profissional no estreitamento do vínculo e em questões relacionadas ao diagnóstico e tratamento do paciente.

No Brasil, o futuro do tratamento da dor depende da realização de novas pesquisas para compreender os fatores que afetam a aceitação inicial e o envolvimento contínuo do uso de aplicativos ou tecnologias digitais para avaliação de pacientes com dor. Isso porque todos os estudos mencionados ilustram a realidade de países desenvolvidos, que difere do contexto de saúde nacional.

## CONSIDERAÇÕES FINAIS

Este estudo pautou-se na literatura científica e na ampla experiência das autoras para desenvolver um protótipo de avaliação de dor do Projeto GerenciaDOR, destinado a um serviço especializado de dor crônica. Ele reforça a importância da atuação dos enfermeiros nessa área e pode motivar o desenvolvimento de novas tecnologias digitais.
